# A placebo-controlled, double-blind, randomized study of recombinant thrombomodulin (ART-123) to prevent oxaliplatin-induced peripheral neuropathy

**DOI:** 10.1007/s00280-020-04135-8

**Published:** 2020-09-23

**Authors:** Masahito Kotaka, Yoji Saito, Takeshi Kato, Hironaga Satake, Akitaka Makiyama, Yasushi Tsuji, Katsunori Shinozaki, Toshiyoshi Fujiwara, Tsunekazu Mizushima, Yasushi Harihara, Naoki Nagata, Naoto Kurihara, Masahiko Ando, Genichi Kusakawa, Takumi Sakai, Yugo Uchida, Mikihiro Takamoto, Saki Kimoto, Ichinosuke Hyodo

**Affiliations:** 1 Gastrointestinal Cancer Center, Sano Hospital, 2-5-1 Shimizugaoka, Tarumi-ku, Kobe-shi, Hyogo 655-0031 Japan; 2 Department of Anesthesiology, Shimane University Faculty of Medicine, 89-1 Enyacho, Izumo City, Shimane 693-8501 Japan; 3 Department of Gastroenterological Surgery, Kansai Rosai Hospital, 3-1-69 Inabaso, Amagasaki-shi, Hyogo 660-8511 Japan; 4 Colorectal Surgery, National Hospital Organization Osaka National Hospital, 2-1-14 Hoenzaka, Chuo-ku, Osaka City, Osaka 540-0006 Japan; 5 Department of Medical Oncology, Kobe City Medical Center General Hospital, 2-1-1 Minatojimaminamimachi, Chuo-ku, Kobe-city, Hyogo 650-0047 Japan; 6 Cancer Treatment Center, Kansai Medical University Hospital, 2-3-1 Shin-machi, Hirakata City, Osaka 573-1191 Japan; 7 Department of Hematology/Oncology, Japan Community Healthcare Organization Kyushu Hospital, 1-8-1 Kishinoura, Yahatanishi-ku, Kitakyusyu-shi, Fukuoka 806-8501 Japan; 8 Cancer Center, Gifu University Hospital, 1-1 Yanagido, Gifu, 501-1194 Japan; 9 Department of Medical Oncology, Tonan Hospital, Kita 4-jo Nishi 7-chome 3-8, Chuo-ku, Sapporo-shi, Hokkaido 060-0004 Japan; 10 Division of Clinical Oncology, Hiroshima Prefectural Hospital, 1-5-54 Ujinakanda, Minami-ku, Hiroshima-shi, Hiroshima 734-8530 Japan; 11 Department of Gastroenterological Surgery, Okayama University Graduate School of Medicine, Dentistry and Pharmaceutical Sciences, 2-5-1 Shikata-cho, Kita-ku, Okayama-city, Okayama 700-8558 Japan; 12 Department of Gastrointestinal Surgery, Osaka University Graduate School of Medicine, 2-2 Yamadaoka, Suita, Osaka 565-0871 Japan; 13 Department of Surgery, NTT Medical Center Tokyo, 5-9-22 Higashi-Gotanda, Shinagawa-ku, Tokyo 141-8625 Japan; 14 Department of Surgery, Kitakyushu General Hospital, 1-1 Higashijono-machi, Kokurakita-ku, Kitakyushu, 802-8517 Japan; 15 Department of Surgery, Nerima General Hospital, 1-24-1 Asahigaoka, Nerima-ku, Tokyo 176-8530 Japan; 16 Center for Advanced Medicine and Clinical Research, Nagoya University Hospital, 65 Tsurumai-cho, Showa-ku, Nagoya, 466-8560 Japan; 17 Clinical Development Center, Asahi Kasei Pharma Corporation, 1-1-2 Yurakucho, Chiyoda-ku, Tokyo 100-0006 Japan; 18 Division of Gastroenterology, University of Tsukuba, 1-1-1 Tennodai, Tsukuba, Ibaraki 305-8577 Japan; 19 Division of Gastrointestinal Medical Oncology, NHO Shikoku Cancer Center, 160 Kou Minami-umemoto, Matsuyama-city, Ehime 791-0280 Japan

**Keywords:** CIPN, Neuropathy, Oxaliplatin, Adjuvant chemotherapy, Colon cancer, Thrombomodulin

## Abstract

**Purpose:**

The purpose of this clinical study was to be the first to explore whether ART-123, a recombinant human soluble thrombomodulin, prevents oxaliplatin-induced peripheral neuropathy (OIPN).

**Methods:**

This randomized, phase IIa trial enrolled stage II/III colon cancer patients who received adjuvant mFOLFOX6 chemotherapy. Participants were randomly allocated to 3 arms in a double-blind manner: placebo (placebo: days 1–3); 1-day ART (ART-123: day 1, placebo: days 2–3); and 3-day ART (ART-123: days 1–3). ART-123 (380 U/kg/day) or placebo was infused intravenously before each 2-week cycle of mFOLFOX6. OIPN was assessed with the Functional Assessment of Cancer Therapy/Gynecological Oncology Group-Neurotoxicity-12 (FACT/GOG-Ntx-12) score by participants and the NCI Common Terminology Criteria for Adverse Events (NCI-CTCAE) by investigators.

**Results:**

Seventy-nine participants (placebo *n* = 28, 1-day ART *n* = 27, 3-day ART *n* = 24) received study drugs. The least-squares mean FACT/GOG-Ntx-12 scores at cycle 12 from the mixed effect model for repeated measures were 28.9 with placebo, 36.3 with 1-day ART (vs. placebo: 7.3 [95% CI 1.9 to12.8, *p* = 0.009]), and 32.3 with 3-day ART (vs. placebo: 3.4 [95% CI −.1 to 9.0, *p* = 0.222]). The cumulative incidence of NCI-CTCAE grade ≥ 2 sensory neuropathy at cycle 12 was 64.3% with placebo, 40.7% with 1-day ART (vs. placebo: −23.5 [95% CI −48.4 to 4.0], *p* = 0.108), and 45.8% with 3-day ART (vs. placebo: −18.5 [95% CI −44.2 to 9.4], *p* = 0.264). Common adverse events were consistent with those reported with mFOLFOX6; no severe bleeding adverse events occurred.

**Conclusion:**

ART-123 showed a potential preventive effect against OIPN with good tolerability. A larger study with 1-day ART is warranted.

NCT02792842, registration date: June 8, 2016

**Electronic supplementary material:**

The online version of this article (10.1007/s00280-020-04135-8) contains supplementary material, which is available to authorized users.

## Introduction

Oxaliplatin is a key drug in the treatment of colorectal cancer and is used in combination with 5-fluorouracil/leucovorin (FOLFOX) or capecitabine for resected stage III colon cancer as adjuvant chemotherapy and for metastatic colorectal cancer as palliative chemotherapy [1–4].

Oxaliplatin-induced peripheral neuropathy (OIPN) is a well-recognized, dose-limiting toxicity. There are two types of neuropathy, acute and chronic (cumulative) neuropathy. Acute neuropathy symptoms including cold allodynia and muscle cramps are generally transient and mild, and they disappear within a few days [5]. Chronic neuropathy is problematic with the oxaliplatin-containing regimen, and its severity is correlated with the cumulative dosage of oxaliplatin [6]. Chronic OIPN is mainly a sensory neuropathy characterized by numbness, paresthesia, and allodynia; motor neuropathy is less frequent. The symptoms of OIPN often limit patients’ daily activities [5, 7, 8]. Chronic OIPN lasts for months or even years after discontinuation of oxaliplatin, and it sometimes worsens transiently for a few months [9–11]. Therefore, there is a need to prevent OIPN in clinical practice, but there are currently no effective agents for OIPN [12, 13]. To relieve only painful OIPN, duloxetine is moderately recommended in the American Society of Clinical Oncology clinical practice guideline [12].

ART-123 is a recombinant human soluble thrombomodulin composed of the extracellular domain of thrombomodulin. In Japan, ART-123 was approved for the treatment of disseminated intravascular coagulation (DIC) in 2008. In previous clinical trials of the treatment of DIC caused by infection, hematological malignancy, and solid tumors, the efficacy and safety of ART-123 at 380 U/kg/day for 6 days were confirmed [14, 15]. ART-123 has an anti-coagulation effect by accelerating the activation of protein C, as well as anti-inflammatory and anti-fibrinolytic effects through activated thrombin-activatable fibrinolysis inhibitor (TAFI) [16–18]. An additional anti-inflammatory effect of ART-123 attributed to direct binding to high-mobility group box 1 protein (HMGB1) and enhancement of its degradation by thrombin has also been reported [19, 20]. Recently, it has been reported that ART-123 prevented oxaliplatin-induced hyperalgesia and allodynia in animal models [21, 22]. The animal study suggested that ART-123 prevents the development of sensory symptoms of OIPN through activation of TAFI and protein C without affecting the anti-tumor activity of oxaliplatin [21]. Moreover, it was reported that an anti-HMGB1-neutralizing antibody prevented oxaliplatin-induced allodynia in animal models [22].

This placebo-controlled, double-blind, randomized, phase IIa clinical study was conducted to explore the preventive effect of ART-123 on OIPN.

## Materials and methods

### Study design and participants

This was a placebo-controlled, randomized, double-blind, phase IIa study to evaluate the efficacy and safety of ART-123 for the prevention of OIPN. This study was registered at Clinicaltrial.gov (ClinicalTrials.gov Identifier: NCT02792842).

Eligible participants had: curatively-resected and histologically-confirmed stage II or III colon cancer, including rectosigmoid cancer, as defined in the 8th edition of the Japanese Classification of Colorectal Carcinoma [23]; age 20–79 years; Eastern Cooperative Oncology Group performance status of 0 or 1; and a plan to receive 12 cycles of postoperative adjuvant chemotherapy with modified FOLFOX6 (mFOLFOX6). Participants with symptomatic peripheral neuropathy, central nervous system damage, any history of chemotherapy/radiotherapy, other malignancy, history of cerebrovascular disorder in the past one year, or major bleeding were excluded.

All participants provided their written, informed consent. This study was conducted in accordance with the Declaration of Helsinki and Good Clinical Practice guidelines. The protocol of this study was approved by the institutional review board of each participating site.

### Randomization and masking

Study drugs consisted of placebo infusion on days 1–3 (placebo arm), ART-123 380 U/kg infusion on day 1 and placebo infusion on days 2–3 (1-day ART arm), and ART-123 380 U/kg infusion on days 1–3 (3-day ART arm). Eligible participants were randomly allocated to the placebo, 1-day ART, and 3-day ART arms in a double-blind manner (1:1:1) by an interactive web response system (IWRS) using random allocation from a computer-generated random number table with permuted blocks of 6 and stratification by site. All persons involved in the study, including participants, investigators and clinical study coordinators performing neuropathy assessments, and sponsors, were blinded to group assignments until unblinding. Lyophilized formulations of ART-123 and placebo with identical appearance were used, and both appearances were identical after dissolution formulations.

### Treatment

Study drug was given intravenously for 30 min once daily on days 1, 2, and 3 in each cycle of mFOLFOX6. On day 1, study drug administration was initiated for 30–120 min before oxaliplatin administration. Study drug was suspended if oxaliplatin treatment was suspended.

The mFOLFOX6 regimen consisted of oxaliplatin 85 mg/m^2^ and levofolinate 200 mg/m^2^ for 2 h, followed by an intravenous bolus of 5-fluorouracil 400 mg/m^2^ and continuous intravenous infusion of 5-fluorouracil 2400 mg/m^2^ for 46 h. A cycle was defined as the period starting from administration of any agent from mFOLFOX6 to the subsequent administration of any agent from mFOLFOX6 and was, thus, typically a two-week period. Dose modification of oxaliplatin due to OIPN was performed based on the sensory and motor neuropathy grades of the National Cancer Institute Common Terminology Criteria for Adverse Events (NCI-CTCAE) version 4.0. Criteria for suspending/reducing the dose of oxaliplatin in association with peripheral neuropathy and restricted concomitant medications, such as pregabalin or gabapentin, are shown in Online Resource 1. Participants who discontinued all agents of the FOLFOX regimen were discontinued from the study (as long as a participant continued one of oxaliplatin, levofolinate, or 5-FU, the participant continued with the remaining study activities).

### Study assessments

OIPN severity was assessed by both participants and investigators. OIPN assessments were performed before administration of any agent, study drug, or chemotherapy, when done on day 1 of each cycle. Participant-reported outcomes were evaluated using the Functional Assessment of Cancer Therapy/Gynecological Oncology Group-Neurotoxicity-12 (FACT/GOG-Ntx-12) version 4.0, which measures the severity and impact of symptoms of neuropathy over the past 7 days [24]. Scores range from 0 to 48, with lower scores indicating more severe neurotoxicity. Participants completed paper questionnaires on days 1 and 8 of each cycle and on days 15 and 43 of cycle 12, with follow-up assessment on the last day (day 43). Clinical research coordinators not involved in the investigators’ evaluations collected questionnaires and entered data into case report forms. Investigators and clinical research coordinators who helped investigators evaluate peripheral neuropathy were blinded to the FACT/GOG-Ntx-12 scores. NCI-CTCAE was used for investigator-reported outcomes of sensory and motor neuropathy; both were assessed every day from day 1 to day 3 of each cycle and on days 15 and 43 of cycle 12. Other adverse events were also assessed by NCI-CTCAE.

No primary endpoint was specified due to the exploratory nature of the study. Exploratory endpoints included the FACT/GOG-Ntx-12 score, cumulative incidence of NCI-CTCAE grade 2 or higher neuropathy, cumulative oxaliplatin dosages to the first grade 2 or higher neuropathy, total cumulative oxaliplatin dosages, and the discontinuation rate of oxaliplatin due to OIPN.

### Statistical analysis

Since this study was exploratory, a precision-based sample size calculation was used. A sample size of 25 participants per treatment arm was calculated to estimate a proportion with a 95% confidence interval (95% CI) whose half-width (the distance between the center of the confidence interval and the upper/lower limit of the confidence interval) is 20%.

The preventive effect of ART-123 on neuropathy was analyzed in all randomly assigned participants who received at least one dose of study drug and oxaliplatin and had a FACT/GOG-Ntx-12 or NCI-CTCAE evaluation at least once after oxaliplatin administration. Safety analyses were performed in participants who received at least one dose of study drug.

The FACT/GOG-Ntx-12 scores were analyzed based on the mixed effect model for repeated measures (MMRM) using all observations including all time points at baseline and each post-baseline visit and the observed case analysis. It was assumed that missing data were missing at random, and missing data were not imputed explicitly in MMRM. The model included the fixed, categorical effects of study treatment, cycle, and study treatment-by-cycle interaction. An unstructured covariance structure was used to model the within-participant errors. The Kenward–Roger approximation was used to estimate denominator degrees of freedom. Least-squares (LS) means were calculated from the MMRM. The *p* value was calculated from the MMRM and the *t*-test in post hoc analyses that were planned after treatment unblinding. The cumulative incidence of participants with grade 2 or higher NCI-CTCAE sensory or motor neuropathy was compared between placebo and 1-day ART arms and between placebo and 3-day ART arms using Fisher’s exact test. Once grade 2 or higher neuropathy was observed in a certain participant, that participant was categorized as grade 2 or higher even if the grade returned to 1 or lower in subsequent cycles. Participants who discontinued the study or whose evaluation data were missing without reaching grade 2 or higher neuropathy were analyzed in two ways: having and not having grade 2 or higher neuropathy. The cumulative oxaliplatin dosages to the first grade 2 or higher neuropathy were analyzed using the Kaplan–Meier method. The difference in the Kaplan–Meier curves of the first grade 2 or higher neuropathy between treatment arms was compared using the log-rank test in post hoc analyses. If there were multiple measurements within a cycle and within a follow-up period, the worst value was used in the analyses to estimate the preventive effect of ART-123 on the worst severity of OIPN. Combined-arm analyses of 1-day ART and 3-day ART arms were also performed in post hoc analyses. All analyses were performed with SAS 9.3. All statistical tests were two-sided, and a *p* value less than 0.05 was considered significant. No adjustment for multiplicity was made.

## Results

### Participants’ characteristics

**Fig. 1:**
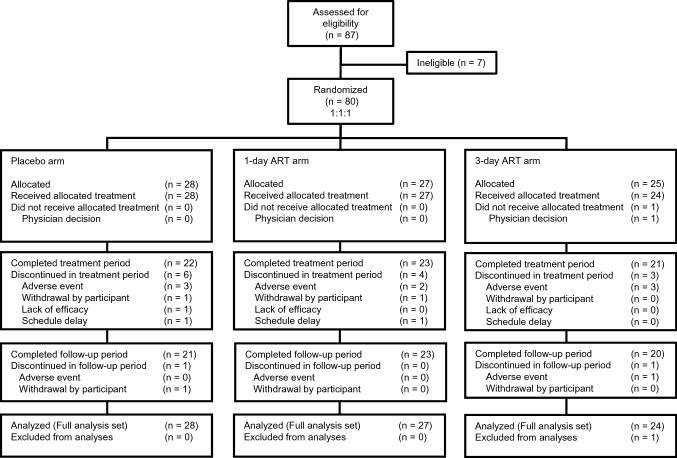
CONSORT diagram. Of the 64 participants who completed follow-up (Placebo: *n* = 21, 1-day ART: *n* = 23, 3-day ART: *n* = 20), 22 (Placebo: *n* = 9, 1-day ART: *n* = 7, 3-day ART: *n* = 6) discontinued oxaliplatin prematurely, but completed treatment and follow-up (day 43 of cycle 12)

Between July 2016 and April 2017, 87 participants were recruited from 11 hospitals in Japan, and 80 participants were randomly assigned to the placebo (*n* = 28), 1-day ART (*n* = 27), and 3-day ART (*n* = 25) arms (Fig. 1). One participant allocated to the 3-day ART arm was unintentionally enrolled in the study due to an operational error of the IWRS and was withdrawn before study drug administration. In total, 79 participants received study drug and were included in the efficacy and safety analyses. Sixty-four participants completed follow-up (Placebo: *n* = 21, 1-day ART: *n* = 23, 3-day ART: *n* = 20), including 22 participants who discontinued oxaliplatin prior to cycle 12, but study assessments were continued (Placebo: *n* = 9, 1-day ART: *n* = 7, 3-day ART: *n* = 6). The baseline characteristics of the participants were well-balanced across arms (Table 1).

**Table 1:** Participants’ baseline characteristics

Characteristic	Placebo *n* = 28	1-day ART *n* = 27	3-day ART *n* = 24
Sex			
Male	16 (57.1)	12 (44.4)	11 (45.8)
Female	12 (42.9)	15 (55.6)	13 (54.2)
Age (years)			
< 65	10 (35.7)	9 (33.3)	11 (45.8)
≥ 65	18 (64.3)	18 (66.7)	13 (54.2)
Median (range)	68.0 (45–79)	68.0 (38–78)	66.0 (32–79)
Weight (kg)			
Median (range)	55.9 (37.4–82.0)	55.3 (41.6–72.4)	58.0 (36.1–93.3)
Body surface area (m^2^)			
Median (range)	1.6 (1.3–2.0)	1.6 (1.3–1.8)	1.6 (1.2–2.1)
Performance status			
0	25 (89.3)	27 (100.0)	24 (100.0)
1	3 (10.7)	0 (0.0)	0 (0.0)
Colon cancer stage			
II	6 (21.4)	3 (11.1)	6 (25.0)
IIIa	16 (57.1)	17 (63.0)	15 (62.5)
IIIb	6 (21.4)	7 (25.9)	3 (12.5)
Diabetes mellitus			
Yes	6 (21.4)	5 (18.5)	4 (16.7)
No	22 (78.6)	22 (81.5)	20 (83.3)
FACT/GOG-Ntx-12			
Mean (SD) score	46.4 (2.0)	46.7 (2.2)	46.3 (2.9)

Data are presented as numbers (%) unless otherwise noted

*ART* recombinant thrombomodulin, *FACT/GOG-Ntx-12* Functional Assessment of Cancer Therapy/Gynecologic Oncology Group-Neurotoxicity-12, *SD* standard deviation

### Participant-reported neuropathy

**Fig. 2:**
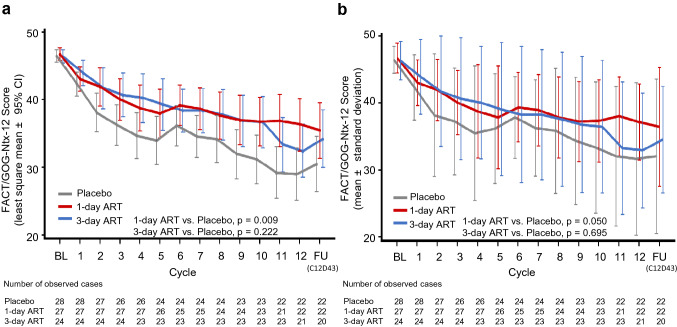
**a** This figure presents the least-squares mean score using MMRM of FACT/GOG-Ntx-12. The *p* value was calculated by MMRM at cycle 12. Error bars represent the 95% confidence interval. **b** This figure presents the mean score using observed case analysis of FACT/GOG-Ntx-12. The *p* values were calculated by *t*-tests at cycle 12. Error bars represent standard deviations. The gray line, red line, and blue line represent the placebo arm, 1-day ART arm, and 3-day ART arm, respectively. *BL* baseline, *FU* follow-up (day 43 of cycle 12), *ART* recombinant thrombomodulin, *FACT/GOG-Ntx-12* Functional Assessment of Cancer Therapy/Gynecologic Oncology Group-Neurotoxicity-12

The LS means of the FACT/GOG-Ntx-12 scores decreased according to the increase of cycle numbers for mFOLFOX6 treatment. The LS means of the FACT/GOG-Ntx-12 scores were 46.4, 46.7, and 46.3 at baseline and 28.9, 36.3, and 32.3 at cycle 12 (day 15) for the placebo, 1-day ART, and 3-day ART arms, respectively (Fig. 2a). The differences in the LS means at cycle 12 were 7.3 (95% CI 1.9 to 12.8, MMRM *p* = 0.009) between the 1-day ART and placebo arms and 3.4 (95% CI −2.1 to 9.0; MMRM *p* = 0.222) between the 3-day ART and placebo arms. Results of the observed case analysis were similar to those of the MMRM analysis (Fig. 2b). These results were also comparable with those in the post hoc combined-arm analyses (Online Resource 2). The means of the FACT/GOG-Ntx-12 scores at days 1 and 8 of each cycle are shown in Online Resource 3.

### Investigator-reported neuropathy

**Fig. 3:**
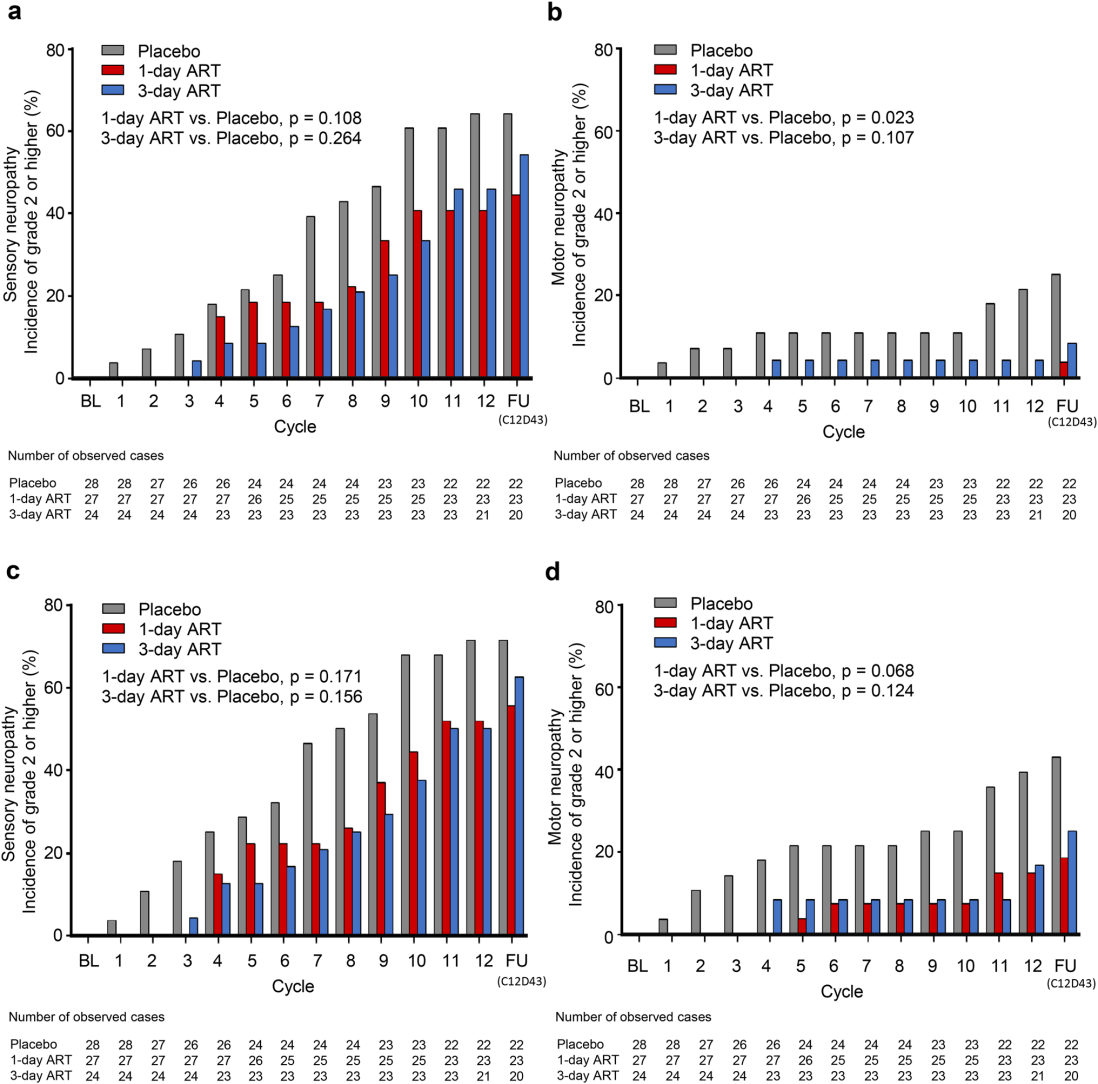
Cumulative incidences of NCI-CTCAE grade 2 or higher sensory neuropathy (**a**), (**c**) and motor neuropathy (**b**), (**d**). Missing grade in participants who discontinued before grade 2 or higher was analyzed as no grade 2 or higher (**a**), (**b**), or as grade 2 or higher (**c**), (**d**). The *p* values were calculated by Fisher’s exact test at cycle 12. The gray bar, red bar, and blue bar represent the placebo arm, 1-day ART arm, and 3-day ART arm, respectively. *BL* baseline, *FU* follow-up (day 43 of cycle 12), *ART* recombinant thrombomodulin

When missing data were analyzed as no grade 2 or higher neuropathy, the cumulative incidences of NCI-CTCAE grade 2 or higher sensory neuropathy at cycle 12 were 64.3% with placebo, 40.7% with 1-day ART (vs. placebo: −23.5 [95% CI − 48.4 to 4.0], *p* = 0.108), and 45.8% with 3-day ART (vs. placebo: −18.5 [95% CI −44.2 to 9.4], *p* = 0.264) (Fig. 3a). The cumulative incidences of grade 2 or higher motor neuropathy at cycle 12 were 21.4% with placebo, 0.0% with 1-day ART (vs. placebo: −21.4 [95% CI −46.0 to 4.6], *p* = 0.023), and 4.2% with 3-day ART (vs. placebo: −17.3 [95% CI −42.9 to 10.3], *p* = 0.107) (Fig. 3b). When missing data were analyzed as grade 2 or higher, both sensory neuropathy and motor neuropathy showed a similar trend (Fig. 3c, d). These results were also comparable with those in the post hoc combined-arm analyses (Online Resource 4). Throughout the entire study, from baseline to follow-up (day 43 of cycle 12), the incidence of grade 1 sensory neuropathy was 32.1%, 55.6%, and 45.8%, the incidence of grade 2 was 64.3%, 40.7%, and 41.7%, and the incidence of grade 3 was 0.0%, 3.7%, and 12.5% in the placebo, 1-day ART, and 3-day ART arms, respectively (Online resource 5a). The incidence of grade 1 motor neuropathy was 10.7%, 25.9%, and 20.8%, the incidence of grade 2 was 17.9%, 3.6%, and 8.3%, and the incidence of grade 3 was 7.1%, 0.0%, and 0.0% in the placebo, 1-day ART, and 3-day ART arms, respectively (Online resource 5b).

**Fig. 4:**
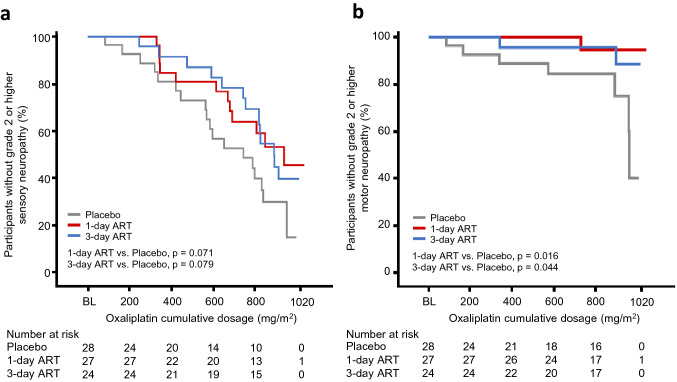
Kaplan–Meier curves of cumulative oxaliplatin dosages to the first NCI-CTCAE grade 2 or higher sensory neuropathy (**a**) and motor neuropathy (**b**). The *p* values were calculated by the log-rank test. The gray line, red line, and blue line represent the placebo arm, 1-day ART arm, and 3-day ART arm, respectively. *BL* baseline, *ART* recombinant thrombomodulin

The median cumulative oxaliplatin dosages to the first grade 2 or higher sensory neuropathy were 747.2 mg/m^2^ (95% CI 566.9 to 840.6 mg/m^2^), 941.0 mg/m^2^ (95% CI 682.5 mg/m^2^ to not reached), and 893.6 mg/m^2^ (95% CI 757.3 mg/m^2^ to not reached) in the placebo, 1-day ART, and 3-day ART arms, respectively (Fig. 4a). The post hoc analysis showed a significant difference between the combined arm and the placebo arm (log-rank test, *p* = 0.032, Online Resource 6a). Results for motor neuropathy showed a similar trend to those of sensory neuropathy (Fig. 4b, Online Resource 6b).

### Administration status of oxaliplatin and use of restricted concomitant medications for OIPN

The median total dosages of oxaliplatin delivered were 819.1 mg/m^2^, 849.2 mg/m^2^, and 920.7 mg/m^2^ for placebo, 1-day ART, and 3-day ART, respectively. The numbers of participants who discontinued oxaliplatin were 15 (53.6%), 11 (40.7%), and 9 (37.5%), of which 9 (32.1%), 4 (14.8%), and 6 (25.0%) were due to OIPN in the placebo, 1-day ART, and 3-day ART arms, respectively. The first dose-reduction of oxaliplatin due to OIPN was observed in cycle 2, cycle 5, and cycle 8 in the placebo, 1-day ART, and 3-day ART arms, respectively (Online Resource 7).

The number of participants who used restricted concomitant medications for OIPN was 4 (14.3%), 4 (14.8%), and 3 (12.5%) in the placebo, 1-day ART, and 3-day ART arms, respectively.

### Safety

The incidence of grade 3 or 4 adverse events was 53.6%, 59.3%, and 66.7% in the placebo, 1-day ART, and 3-day ART arms, respectively (Table 2). Serious adverse events were observed in 2 (7.1%), 5 (18.5%), and 3 (12.5%) in the placebo, 1-day ART, and 3-day ART arms, respectively. All serious adverse events were assessed to be unrelated to study drug. Common adverse events were consistent with those reported with mFOLFOX6. Because of the anti-coagulation effect of ART-123, bleeding-related adverse events were of particular interest. The number of bleeding adverse events is shown in Table 2. Grade 2 hematuria occurred in one participant in the 1-day ART arm, and all other bleeding adverse events were grade 1. ART-123-related bleeding adverse events as judged by investigators were epistaxis in the 3-day ART arm (*n* = 2) and purpura in the 1-day ART arm (*n* = 1).

**Table 2:** Adverse events

	Placebo (*n* = 28)		1-day ART (*n* = 27)		3-day ART (*n* = 24)	
	All grades	Grade 3/4	All grades	Grade 3/4	All grades	Grade 3/4
Overall AEs	28 (100.0)	15 (53.6)	27 (100.0)	16 (59.3)	24 (100.0)	16 (66.7)
Most common AEs						
Neutrophil count decreased	12 (42.9)	9 (32.1)	13 (48.1)	8 (29.6)	16 (66.7)	11 (45.8)
Malaise	17 (60.7)	0 (0.0)	11 (40.7)	0 (0.0)	12 (50.0)	0 (0.0)
Nausea	9 (32.1)	0 (0.0)	12 (44.4)	1 (3.7)	9 (37.5)	0 (0.0)
Inappetence	12 (42.9)	1 (3.6)	8 (29.6)	0 (0.0)	8 (33.3)	0 (0.0)
Dysgeusia	7 (25.0)	0 (0.0)	7 (25.9)	0 (0.0)	8 (33.3)	0 (0.0)
Fatigue	7 (25.0)	1 (3.6)	5 (18.5)	0 (0.0)	8 (33.3)	0 (0.0)
Constipation	6 (21.4)	0 (0.0)	7 (25.9)	0 (0.0)	4 (16.7)	0 (0.0)
Diarrhea	5 (17.9)	0 (0.0)	5 (18.5)	0 (0.0)	5 (20.8)	1 (4.2)
Alopecia	4 (14.3)	0 (0.0)	6 (22.2)	0 (0.0)	2 (8.3)	0 (0.0)
Mucositis oral	3 (10.7)	1 (3.6)	4 (14.8)	0 (0.0)	5 (20.8)	0 (0.0)
White blood cell count decreased	1 (3.6)	0 (0.0)	6 (22.2)	1 (3.7)	3 (12.5)	0 (0.0)
Bleeding AEs	2 (7.1)	0 (0.0)	3 (11.1)	0 (0.0)	6 (25.0)	0 (0.0)
Epistaxis	1 (3.6)	0 (0.0)	2 (7.4)	0 (0.0)	3 (12.5)	0 (0.0)
Implant site hemorrhage	0 (0.0)	0 (0.0)	0 (0.0)	0 (0.0)	2 (8.3)	0 (0.0)
Bronchopulmonary hemorrhage	1 (3.6)	0 (0.0)	0 (0.0)	0 (0.0)	0 (0.0)	0 (0.0)
Hematuria	0 (0.0)	0 (0.0)	1 (3.7)	0 (0.0)	0 (0.0)	0 (0.0)
Hyposphagma	0 (0.0)	0 (0.0)	0 (0.0)	0 (0.0)	1 (4.2)	0 (0.0)
Purpura	0 (0.0)	0 (0.0)	1 (3.7)	0 (0.0)	0 (0.0)	0 (0.0)

All data are shown as Nos. (%)

*AE* adverse event, *ART* recombinant thrombomodulin

## Discussion

This was the first clinical study to explore the efficacy and safety of ART-123 for the prevention of OIPN. ART-123 significantly inhibited the decrease of FACT/GOG-Ntx-12 scores in the 1-day ART arm. ART-123 tended to decrease the cumulative incidence of NCI-CTCAE grade 2 or higher sensory neuropathy caused by oxaliplatin treatment, although not significantly. No clear advantages in efficacy of the 3-day ART arm were observed compared to the 1-day ART arm. ART-123 was well-tolerated in participants who received adjuvant mFOLFOX6 chemotherapy for colon cancer.

According to ACTTION (Analgesic, Anesthetic, and Addiction Clinical Trial Translations, Innovations, Opportunities and Networks) recommendations, a participant-reported outcome should always be included in clinical studies of chemotherapy-induced peripheral neuropathy (CIPN) prevention [25]. The FACT/GOG-Ntx-12 was selected as a participant-reported outcome instrument for assessment of OIPN, because the FACT/GOG-Ntx scale is a reliable, valid, and widely-used CIPN assessment tool, including for participants with colorectal cancer treated by oxaliplatin [24, 26–28]. The changes in the FACT/GOG-Ntx scores in the placebo arm of the present study were similar to those in other reported clinical trials [29–31]. Approximately 4-point differences of the LS mean scores between the ART and placebo arms were continuously observed in the latter half of the treatment cycles. A 4-point difference in the FACT/GOG-Ntx-12 scores is reported to be clinically meaningful [24]. Moreover, considering that the median cumulative oxaliplatin dosages were around 820 mg/m^2^ in the placebo arm and over 840 mg/m^2^ in both ART arms, the higher score of the FACT/GOG-Ntx-12 of the 1-day ART arm compared to placebo was not due to less oxaliplatin delivery and suggests the possibility that ART-123 prevents OIPN.

NCI-CTCAE Grade 2 or higher sensory neuropathy was reported to occur in 40–60% of participants receiving FOLFOX at an oxaliplatin dosage level of 700–800 mg/m^2^ in previous studies [29, 32, 33]. The present results of the placebo arm were consistent with those reports.

Study drug was given on days 1, 2, and 3 in each cycle of mFOLFOX6. The dose regimen was determined based on a non-clinical study using a rat model of OIPN (data not shown) showing that a seven-day consecutive dosing regimen through days 1–7 provided a more potent preventive effect than a single dosing regimen on day 1 against oxaliplatin-induced hyperalgesia. Considering the inconvenience of the participants and site staff to administer study drug, the three-day dosing regimen (3-day ART arm) was established as the acceptable maximum dose frequency for sites based on a preliminary feasibility web-based survey. On the other hand, from the practical standpoint, a single dosing regimen (1-day ART) on day 1 of each cycle is preferred, given that patients generally visit one day in a cycle in clinical practice. In the present study, the preventive effects in the 3-day ART arm were not greater than those in the 1-day ART arm. These results suggest that administration of ART-123 prior to oxaliplatin on day 1 was the predominant contributor to reducing the severity and incidence of OIPN in this human study. One possible explanation for this discrepancy between the non-clinical and clinical findings is the shorter half-life of ART-123 in rats than in humans, but the details are not clear [34, 35].

The safety profile of ART-123 was generally mild and well-tolerated in participants with resected colon cancer, as expected. Although some adverse events occurred in all participants, they were similar to those reported in past studies using FOLFOX [1, 31, 36]. Particular attention was paid to bleeding adverse events because ART-123 has an anti-coagulation effect. For participants with DIC, bleeding-related adverse events, such as microscopic hematuria, were reported in the previous clinical trial [15]. In the present study, the most frequently observed bleeding adverse event was epistaxis. Only one grade 2 bleeding event, hematuria, occurred in the 1-day ART arm. In this participant, a thromboembolic event in the lower limbs occurred after 2 cycles of mFOLFOX6, and apixaban treatment was started. Then, grade 2 hematuria occurred before cycle 5, but disappeared soon after stopping apixaban. Therefore, this hematuria was assessed to be unrelated to study drugs. Further investigation is needed to demonstrate to what extent the bleeding risk of ART-123 increases when used concomitantly with anticoagulants.

The maximum plasma concentrations achieved with single- or three-day administration of ART-123 were 1001 ± 170 ng/mL and 1526 ± 269 ng/mL, respectively, in this study. According to previous reports, activation of TAFI and protein C by ART-123 was expected at a concentration range of 100–3200 ng/mL, based on data from an in vitro non-clinical study using human plasma [17, 18]. In addition, administration of ART-123, activated TAFI homolog, and exogenous human-activated protein C prevented oxaliplatin-induced hyperalgesia and allodynia in animal models [21]. Therefore, it is hypothesized that ART-123 prevents OIPN by promoting activation of TAFI and protein C. However, the exact mechanisms and the relationships between OIPN and TAFI and/or APC have not been elucidated.

This study has some limitations. First, multiple tests were performed with no adjustment for multiplicity because the study was exploratory, and the small sample size limited the power of the study. Second, the follow-up period after study drug completion was not long enough. Symptoms of OIPN are known to continue or even worsen over 3 months after completion of chemotherapy [7, 29]. Third, although it was confirmed that ART-123 did not have an impact on the anti-tumor effects of oxaliplatin in preclinical studies, the present study lacked data about whether ART-123 affected the anti-tumor activity of human chemotherapy [21]. Fourth, since no objective measurement was available, no information was obtained regarding the effect of ART-123 on sensory nerve damage or function. Last, this study was conducted only in Japan, which may limit generalization of its results. These limitations should be resolved in future global studies with larger sample sizes and longer follow-up periods.

In conclusion, this phase IIa exploratory study suggests that the recombinant human soluble thrombomodulin ART-123 has a potential preventive effect against OIPN with good tolerability. Further studies are warranted based on these results.

## Electronic supplementary material

Below is the link to the electronic supplementary material.

Supplementary file1 (PDF 254 kb)

Supplementary file2 (PDF 325 kb)

Supplementary file3 (PDF 340 kb)

Supplementary file4 (PDF 284 kb)

Supplementary file5 (PDF 255 kb)

Supplementary file6 (PDF 270 kb)

Supplementary file7 (PDF 262 kb)

## Data Availability

The datasets generated during the current study are not publicly available because the informed consent form signed by the participants did not address an individual data sharing statement.
