# Supplementary material for: A placebo-controlled, double-blind, randomized study of recombinant thrombomodulin (ART-123) to prevent oxaliplatin-induced peripheral neuropathy
Source: Cancer Chemother Pharmacol. 2020 Sep 23;86(5):607–18. doi: 10.1007/s00280-020-04135-8 (PMC7561567; doi:10.1007/s00280-020-04135-8)
Supplement: Supplementary file 1 — Supplementary file1 (PDF 254 kb) [file 280_2020_4135_MOESM1_ESM.pdf]

Title : A placebo-controlled, double-blind, randomized study of recombinant thrombomodulin (ART-123) to prevent oxaliplatin-induced peripheral neuropathy
Journal : *Cancer Chemotherapy and Pharmacology*
Corresponding author: Masahito Kotaka
Gastrointestinal Cancer Center, Sano Hospital
tomomakotaka6410@yahoo.co.jp

Online resource 1

Criteria for suspending or reducing the dose of oxaliplatin in association with peripheral neuropathy

Criteria for suspending or reducing the dose of oxaliplatin in association with peripheral neuropathy were set as follows:
When grade 2 neuropathy occurred and grade 1 neuropathy still persisted immediately before oxaliplatin administration, reduction of the oxaliplatin dose to 75 mg/m^2 was allowed at the investigator’s discretion.
When grade 2 neuropathy was observed immediately before oxaliplatin administration, the oxaliplatin dose was reduced to 75 mg/m^2, or oxaliplatin was postponed.
Oxaliplatin was stopped when grade 3 neuropathy occurred.

Restricted concomitant medication

The following concomitant medications were prohibited except when grade 2 or higher peripheral sensory or motor neuropathy occurred: pregabalin, gabapentin, goshajinkigan (Japanese herbal medicine), serotonin/noradrenaline reuptake inhibitors, and narcotics.
