# Supplementary material for: A placebo-controlled, double-blind, randomized study of recombinant thrombomodulin (ART-123) to prevent oxaliplatin-induced peripheral neuropathy
Source: Cancer Chemother Pharmacol. 2020 Sep 23;86(5):607–18. doi: 10.1007/s00280-020-04135-8 (PMC7561567; doi:10.1007/s00280-020-04135-8)
Supplement: Supplementary file 2 — Supplementary file2 (PDF 325 kb) [file 280_2020_4135_MOESM2_ESM.pdf]

Title : A placebo-controlled, double-blind, randomized study of recombinant thrombomodulin (ART-123) to prevent oxaliplatin-induced peripheral neuropathy
Journal : *Cancer Chemotherapy and Pharmacology*
Corresponding author: Masahito Kotaka
Gastrointestinal Cancer Center, Sano Hospital
tomomakotaka6410@yahoo.co.jp

Online resource 2 FACT/GOG-NTX-12 scores (combined ART arm)

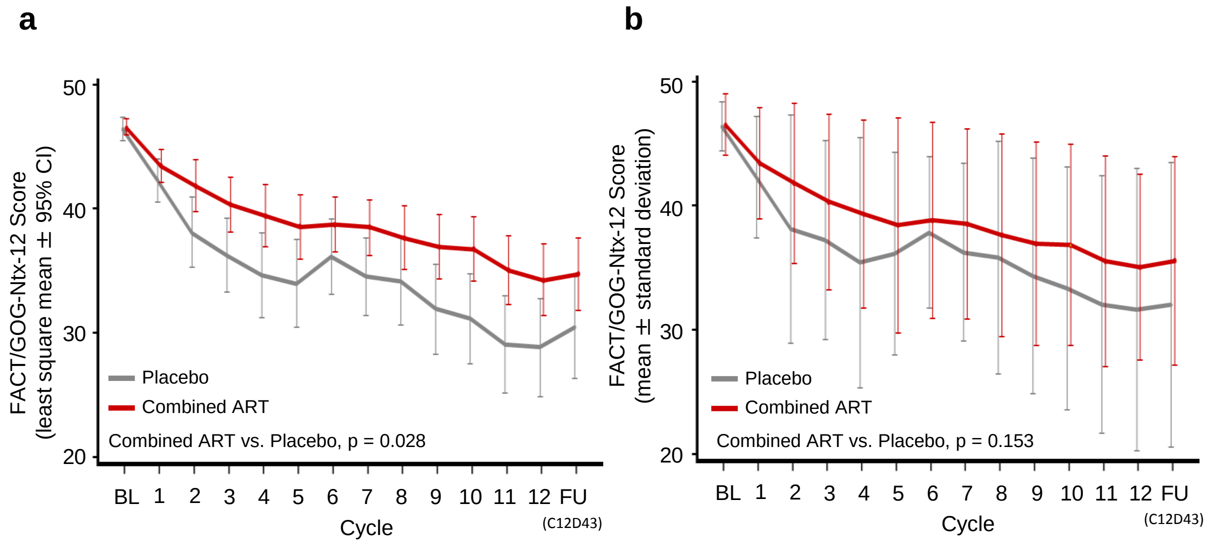

(a) No. ; observed case

Placebo	28	28	27	26	26	24	24	24	24	23	23	22	22	22
Combined ART	51	51	51	51	50	49	48	48	47	47	46	44	43	42

(b) No. ; observed case

Placebo	28	28	27	26	26	24	24	24	24	23	23	22	22	22
Combined ART	51	51	51	51	50	49	48	48	47	47	46	44	43	42

The 1-day ART and 3-day ART arms are combined into one arm (combined ART arm) as a post hoc analysis. (a) This figure presents the least-squares mean score using MMRM of FACT/GOG-Ntx-12. The *P* value was calculated by MMRM at cycle 12. Error bars represent the 95% confidence intervals. (b) This figure presents the mean score using observed case analysis of FACT/GOG-Ntx-12. The *p* values were calculated by *t*-tests at cycle 12. Error bars represent standard deviations. The gray line represents the placebo arm, and the red line represents the combined ART arm. BL, baseline; FU, follow-up (day 43 of cycle 12); ART, recombinant thrombomodulin; FACT/GOG-Ntx-12, Functional Assessment of Cancer Therapy/Gynecologic Oncology Group-Neurotoxicity-12
