# Supplementary material for: A placebo-controlled, double-blind, randomized study of recombinant thrombomodulin (ART-123) to prevent oxaliplatin-induced peripheral neuropathy
Source: Cancer Chemother Pharmacol. 2020 Sep 23;86(5):607–18. doi: 10.1007/s00280-020-04135-8 (PMC7561567; doi:10.1007/s00280-020-04135-8)
Supplement: Supplementary file 3 — Supplementary file3 (PDF 340 kb) [file 280_2020_4135_MOESM3_ESM.pdf]

Title : A placebo-controlled, double-blind, randomized study of recombinant thrombomodulin (ART-123) to prevent oxaliplatin-induced peripheral neuropathy
Journal : *Cancer Chemotherapy and Pharmacology*
Corresponding author: Masahito Kotaka
Gastrointestinal Cancer Center, Sano Hospital
tomomakotaka6410@yahoo.co.jp

Online resource 3 FACT/GOG-Ntx-12 scores at days 1 and 8 of each cycle

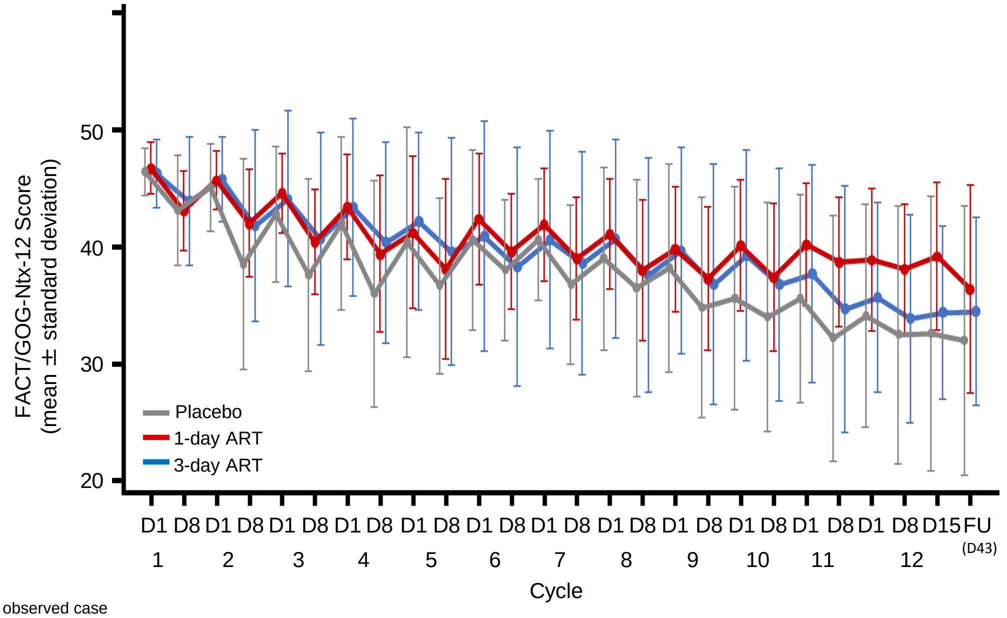

No. ; observed case	C1 D1	C1 D8	C2 D1	C2 D8	C3 D1	C3 D8	C4 D1	C4 D8	C5 D1	C5 D8	C6 D1	C6 D8	C7 D1	C7 D8	C8 D1	C8 D8	C9 D1	C9 D8	C10 D1	C10 D8	C11 D1	C11 D8	C12 D1	C12 D8	C12 D15	FU (D43)
Placebo	28	28	27	27	26	26	26	26	24	24	24	24	24	24	24	24	23	23	23	23	22	22	22	22	22	22
1-day ART-123	27	27	27	27	27	27	27	27	26	26	25	25	25	25	25	24	24	24	24	23	21	21	21	21	22	22
3-day ART-123	24	24	24	24	24	24	23	23	23	23	23	23	23	23	23	23	23	23	23	23	23	23	21	21	21	20

The mean score using observed case analysis of FACT/GOG-Ntx-12 at days 1 and 8 of each cycle. Error bars represent standard deviations. The gray line, red line, and blue line represent the placebo arm, 1-day ART arm, and 3-day ART arm, respectively. BL, baseline; FU, follow-up (day 43 of cycle 12); D, Day; ART, recombinant thrombomodulin; FACT/GOG-NTX-12, Functional Assessment of Cancer Therapy/Gynecologic Oncology Group-Neurotoxicity-12
