# Supplementary material for: A placebo-controlled, double-blind, randomized study of recombinant thrombomodulin (ART-123) to prevent oxaliplatin-induced peripheral neuropathy
Source: Cancer Chemother Pharmacol. 2020 Sep 23;86(5):607–18. doi: 10.1007/s00280-020-04135-8 (PMC7561567; doi:10.1007/s00280-020-04135-8)
Supplement: Supplementary file 4 — Supplementary file4 (PDF 284 kb) [file 280_2020_4135_MOESM4_ESM.pdf]

Title : A placebo-controlled, double-blind, randomized study of recombinant thrombomodulin (ART-123) to prevent oxaliplatin-induced peripheral neuropathy
Journal : *Cancer Chemotherapy and Pharmacology*
Corresponding author: Masahito Kotaka
Gastrointestinal Cancer Center, Sano Hospital
tomomakotaka6410@yahoo.co.jp

Online resource 4 Cumulative incidence of NCI-CTCAE grade 2 or higher neuropathy (combined ART arm)

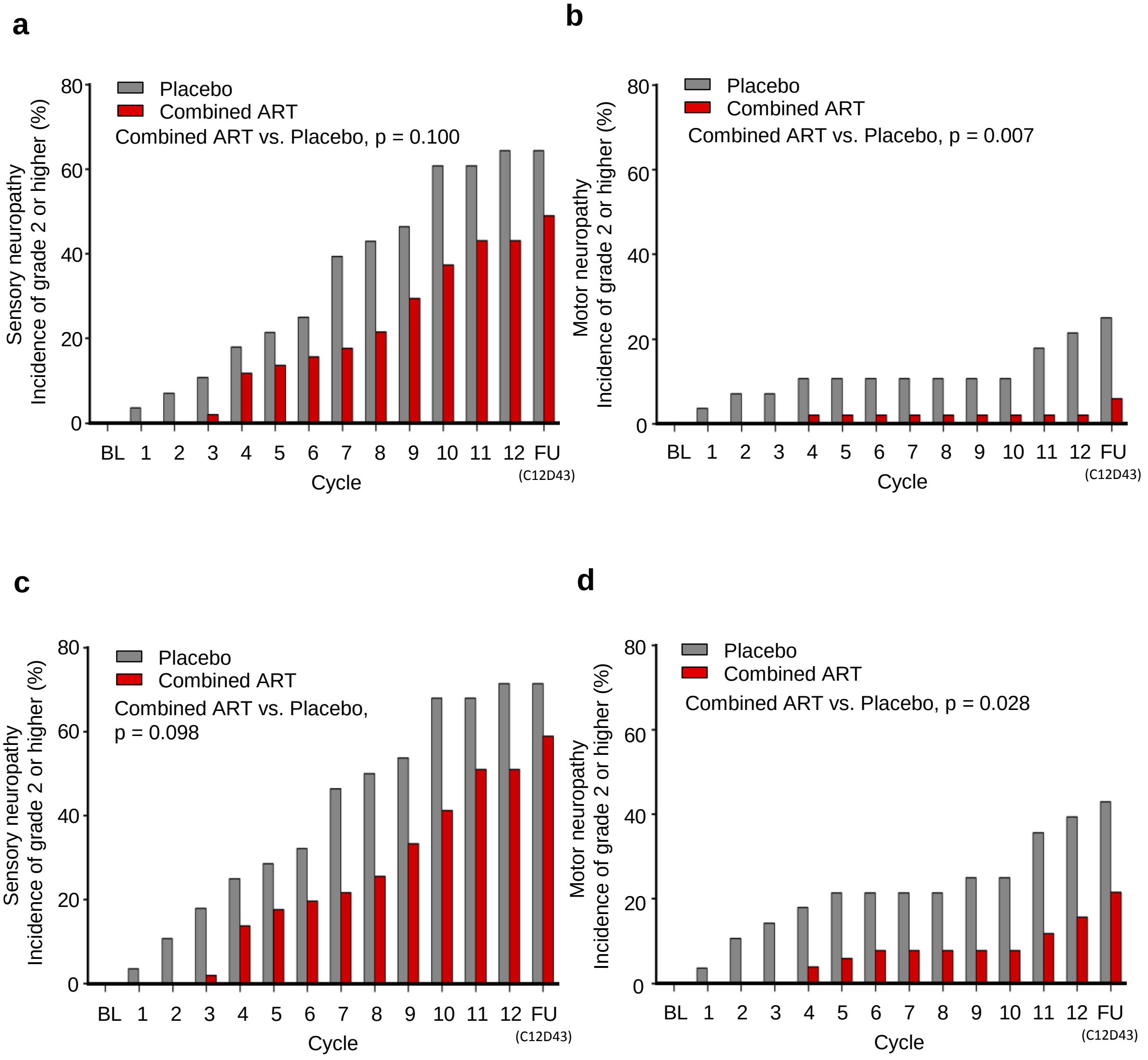

Cumulative incidence of NCI-CTCAE grade 2 or higher sensory neuropathy (a), (c) and motor neuropathy (b), (d). The 1-day ART and 3-day ART arms are combined into one arm (combined ART arm) as a post hoc analysis. Missing grade in participants who discontinued before grade 2 or higher was analyzed as no grade 2 or higher (a), (b), or as grade 2 or higher (c), (d). The *p* values were calculated from Fisher's exact test at cycle 12. The gray bar represents the placebo arm, and the red bar represents the combined arm. BL, baseline; FU, follow-up (day 43 of cycle 12); ART, recombinant thrombomodulin
