# Supplementary material for: A placebo-controlled, double-blind, randomized study of recombinant thrombomodulin (ART-123) to prevent oxaliplatin-induced peripheral neuropathy
Source: Cancer Chemother Pharmacol. 2020 Sep 23;86(5):607–18. doi: 10.1007/s00280-020-04135-8 (PMC7561567; doi:10.1007/s00280-020-04135-8)
Supplement: Supplementary file 5 — Supplementary file5 (PDF 255 kb) [file 280_2020_4135_MOESM5_ESM.pdf]

Title : A placebo-controlled, double-blind, randomized study of recombinant thrombomodulin (ART-123) to prevent oxaliplatin-induced peripheral neuropathy
Journal : *Cancer Chemotherapy and Pharmacology*
Corresponding author: Masahito Kotaka
Gastrointestinal Cancer Center, Sano Hospital
tomomakotaka6410@yahoo.co.jp

Online resource 5 The incidence of NCI-CTCAE each grade (Grades 1, 2, and 3) sensory and motor neuropathy: worst grade through the study

a

Sensory neuropathy	Placebo *n* = 28	1-day ART *n* = 27	3-day ART *n* = 24
None	1 (3.6)	0 (0.0)	0 (0.0)
Grade 1	9 (32.1)	15 (55.6)	11 (45.8)
Grade 2	18 (64.3)	11 (40.7)	10 (41.7)
Grade 3	0 (0.0)	1 (3.6)	3 (12.5)

Note. All data are shown as No. (%). ART, recombinant thrombomodulin

b

Motor neuropathy	Placebo *n* = 28	1-day ART *n* = 27	3-day ART *n* = 24
None	18 (64.3)	19 (70.4)	17 (70.8)
Grade 1	3 (10.7)	7 (25.9)	5 (20.8)
Grade 2	5 (17.9)	1 (3.7)	2 (8.3)
Grade 3	2 (7.1)	0 (0.0)	0 (0.0)

Note. All data are shown as No. (%). ART, recombinant thrombomodulin
