# Supplementary material for: A placebo-controlled, double-blind, randomized study of recombinant thrombomodulin (ART-123) to prevent oxaliplatin-induced peripheral neuropathy
Source: Cancer Chemother Pharmacol. 2020 Sep 23;86(5):607–18. doi: 10.1007/s00280-020-04135-8 (PMC7561567; doi:10.1007/s00280-020-04135-8)
Supplement: Supplementary file 6 — Supplementary file6 (PDF 270 kb) [file 280_2020_4135_MOESM6_ESM.pdf]

Title : A placebo-controlled, double-blind, randomized study of recombinant thrombomodulin (ART-123) to prevent oxaliplatin-induced peripheral neuropathy
Journal : *Cancer Chemotherapy and Pharmacology*
Corresponding author: Masahito Kotaka
Gastrointestinal Cancer Center, Sano Hospital
tomomakotaka6410@yahoo.co.jp

Online resource 6 Kaplan-Meier curves of cumulative oxaliplatin dosages to the first NCI-CTCAE grade 2 or higher neuropathy

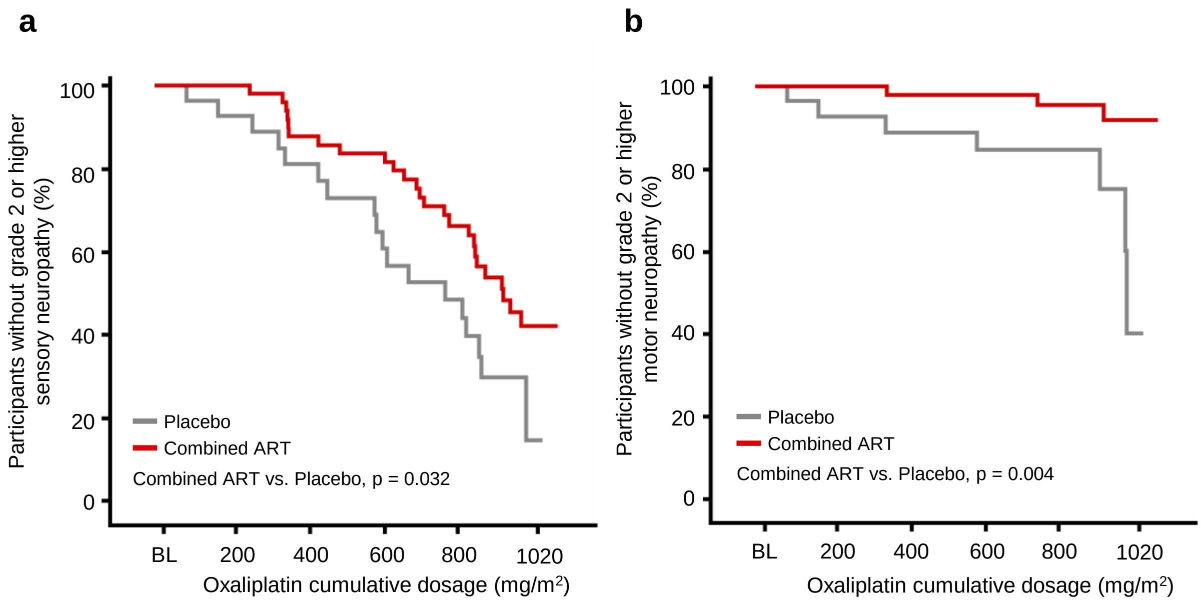

(a)

No. at risk						
Placebo	28	24	20	14	10	0
Combined ART	51	51	43	39	28	1

(b)

No. at risk						
Placebo	28	24	21	18	16	0
Combined ART	51	51	48	44	34	1

Kaplan-Meier curves of cumulative oxaliplatin dosages to the first NCI-CTCAE grade 2 or higher sensory neuropathy (a) and motor neuropathy (b) in a post hoc analysis. The 1-day ART and 3-day ART arms are combined into one arm (combined ART arm). The *p* values were calculated using the log-rank test. The gray line represents the placebo arm, and the red line represents the combined arm. BL, baseline; ART, recombinant thrombomodulin
