# Supplementary material for: A placebo-controlled, double-blind, randomized study of recombinant thrombomodulin (ART-123) to prevent oxaliplatin-induced peripheral neuropathy
Source: Cancer Chemother Pharmacol. 2020 Sep 23;86(5):607–18. doi: 10.1007/s00280-020-04135-8 (PMC7561567; doi:10.1007/s00280-020-04135-8)
Supplement: Supplementary file 7 — Supplementary file7 (PDF 262 kb) [file 280_2020_4135_MOESM7_ESM.pdf]

Title : A placebo-controlled, double-blind, randomized study of recombinant thrombomodulin (ART-123) to prevent oxaliplatin-induced peripheral neuropathy
Journal : *Cancer Chemotherapy and Pharmacology*
Corresponding author: Masahito Kotaka
Gastrointestinal Cancer Center, Sano Hospital
tomomakotaka6410@yahoo.co.jp

Online resource 7 Reduction of oxaliplatin dose at each cycle

	Placebo *n* = 28			1-day ART *n* = 27			3-day ART *n* = 24		
		Reason			Reason			Reason	
	Total n (%)	OIPN n (%)	Non-OIPN n (%)	Total n (%)	OIPN n (%)	Non-OIPN n (%)	Total n (%)	OIPN n (%)	Non-OIPN n (%)
C1	0 (0.0)	0 (0.0)	0 (0.0)	0 (0.0)	0 (0.0)	0 (0.0)	0 (0.0)	0 (0.0)	0 (0.0)
C2	4 (14.3)	1 (3.6)	3 (10.7)	2 (7.4)	0 (0.0)	2 (7.4)	2 (8.3)	0 (0.0)	2 (8.3)
C3	5 (17.9)	1 (3.6)	4 (14.3)	7 (25.9)	0 (0.0)	7 (25.9)	4 (16.7)	0 (0.0)	4 (16.7)
C4	7 (25.0)	1 (3.6)	6 (21.4)	7 (25.9)	0 (0.0)	7 (25.9)	3 (12.5)	0 (0.0)	3 (12.5)
C5	8 (28.6)	1 (3.6)	7 (25.0)	11 (40.7)	2 (7.4)	9 (33.3)	4 (16.7)	0 (0.0)	4 (16.7)
C6	12 (42.9)	1 (3.6)	11 (39.3)	10 (37.0)	1 (3.7)	9 (33.3)	6 (25.0)	0 (0.0)	6 (25.0)
C7	11 (39.3)	1 (3.6)	10 (35.7)	12 (44.4)	1 (3.7)	11 (40.7)	7 (29.2)	0 (0.0)	7 (29.2)
C8	13 (46.4)	2 (7.1)	11 (39.3)	13 (48.1)	2 (7.4)	11 (40.7)	8 (33.3)	1 (4.2)	7 (29.2)
C9	13 (46.4)	3 (10.7)	10 (35.7)	15 (55.6)	4 (14.8)	11 (40.7)	9 (37.5)	3 (12.5)	6 (25.0)
C10	13 (46.4)	3 (10.7)	10 (35.7)	10 (37.0)	2 (7.4)	8 (29.6)	10 (41.7)	3 (12.5)	7 (29.2)
C11	11 (39.3)	3 (10.7)	8 (28.6)	12 (44.4)	4 (14.8)	8 (29.6)	10 (41.7)	4 (16.7)	6 (25.0)
C12	10 (35.7)	3 (10.7)	7 (25.0)	10 (37.0)	4 (14.8)	6 (22.2)	6 (25.0)	2 (8.3)	4 (16.7)

Note. ART, recombinant thrombomodulin; OIPN, oxaliplatin-induced peripheral neuropathy; C, cycle
